# The Rewarding Aspects of Music Listening Are Related to Degree of Emotional Arousal

**DOI:** 10.1371/journal.pone.0007487

**Published:** 2009-10-16

**Authors:** Valorie N. Salimpoor, Mitchel Benovoy, Gregory Longo, Jeremy R. Cooperstock, Robert J. Zatorre

**Affiliations:** 1 Montreal Neurological Institute, McGill University, Montreal, Quebec, Canada; 2 Department of Psychology, McGill University, Montreal, Quebec, Canada; 3 Centre for Interdisciplinary Research on Music Media and Technology, Montreal, Quebec, Canada; 4 International Laboratory for Brain, Music and Sound Research (BRAMS), Montreal, Quebec, Canada; 5 Centre for Intelligent Machines, McGill University, Montreal, Quebec, Canada; Victoria University of Wellington, New Zealand

## Abstract

**Background:**

Listening to music is amongst the most rewarding experiences for humans. Music has no functional resemblance to other rewarding stimuli, and has no demonstrated biological value, yet individuals continue listening to music for pleasure. It has been suggested that the pleasurable aspects of music listening are related to a change in emotional arousal, although this link has not been directly investigated. In this study, using methods of high temporal sensitivity we investigated whether there is a systematic relationship between dynamic increases in pleasure states and physiological indicators of emotional arousal, including changes in heart rate, respiration, electrodermal activity, body temperature, and blood volume pulse.

**Methodology:**

Twenty-six participants listened to self-selected intensely pleasurable music and “neutral” music that was individually selected for them based on low pleasure ratings they provided on other participants' music. The “chills” phenomenon was used to index intensely pleasurable responses to music. During music listening, continuous real-time recordings of subjective pleasure states and simultaneous recordings of sympathetic nervous system activity, an objective measure of emotional arousal, were obtained.

**Principal Findings:**

Results revealed a strong positive correlation between ratings of pleasure and emotional arousal. Importantly, a dissociation was revealed as individuals who did not experience pleasure also showed no significant increases in emotional arousal.

**Conclusions/Significance:**

These results have broader implications by demonstrating that strongly felt emotions could be rewarding in themselves in the absence of a physically tangible reward or a specific functional goal.

## Introduction

Why is music pleasurable? It is simply a sequence of tones. Yet music has been present in every known human culture as far back as history dates. Although there are various theories as to why music may have developed (for a review see [1]), the intense degree of pleasure associated with listening to music remains a mystery. The conundrum lies in the fact that there are no direct functional similarities between music and other pleasure-producing stimuli: it has no clearly established biological value (cf., food, love, and sex), no tangible basis (cf., pharmacological drugs and monetary rewards), and no known addictive properties (cf., gambling and nicotine). Despite this, music is consistently ranked amongst the top ten things that individuals find highly pleasurable [2], and it plays a ubiquitous and important role in most people's lives.

One prominent theory is that music asserts its effects through influencing emotions [3]–[5]. It follows from this that music may evoke or enhance emotions, and that emotion in itself could be rewarding. Empirical evidence linking music and emotional arousal comes from subjective reports that “modifying emotions” is reported as the top reason why people listen to music [6]–[9], as well as through objective measures of physiological changes in the body in a direction indicative of emotional arousal during music listening [8], [10]–[13]. Emotion, by definition, involves a physiological component [14]–[16]. More specifically, emotional arousal is physiologically marked by increased activity of the sympathetic branch of the autonomic nervous system, without voluntary control. The connection between emotional arousal and sympathetic nervous system activity has been well-established [17]–[19]. This connection has also been substantiated with music stimuli [8], [20]. It should be noted, however, that while physiological arousal is a reliable indicator of the “arousal” component of emotions, it is more controversial with respect to detecting the “valence” dimension of emotions. This concern does not present a problem here, as we are only interested in emotions with positive valence.

Despite the established link between music listening and emotional arousal, it is not clear whether this arousal underlies the pleasurable aspects of music, and there is little empirical evidence to suggest that emotional arousal is directly related to music's rewarding properties. The aim of our study was to test this theory by systematically examining the relationship between emotional arousal and pleasure during music listening. Pleasure is a construct that refers to a subjective state, and implies that the associated behavior is rewarding and likely to be repeated. The broader implication of this experiment is to understand why humans feel pleasure from listening to music, and why this behavior continues to be repeated despite any apparent functional significance. One way to examine this question would be to manipulate the degree of pleasure experienced in response to music listening on a continuum, and examine the underlying changes in emotional arousal. This represents a challenge as the range of such a manipulation would have to be large enough to produce sufficient variability for reliable results. In other words, a mild change in mood would not suffice; participants would have to experience “no pleasure” on one end of the spectrum and “extreme pleasure” on the other (also see [21]). Since musical preferences vary widely [11], [22], [23], this would be an extremely difficult task to achieve with a single set of experimenter-selected musical samples. Thus, we used participant-selected music. However, this, in turn, implies that different stimuli would be used for different individuals, not allowing one to accurately conclude whether any observed changes in physiology are specific to acoustical parameters of the music (e.g., changes in tempo, pitch, harmony, etc.), or to a change in subjective state. For example, some studies suggest that changes in tempo lead to corresponding changes in respiration rate [24], [25]. If it is also the case that a participant begins to enjoy a piece of music more as its tempo increases, one would not be able to conclude whether any corresponding changes in respiration rate are attributable to increases in pleasure states, or a bottom-up physiological reaction to psychoacoustical features of the music. As such, it is necessary to distinguish between any changes in physiology that may be caused by the stimulus more generally from those that are reflective of individualized emotional response to that stimulus. Here, we developed a paradigm that separates these two processes. First, participants were asked to provide self-selected musical pieces to which they experience intense pleasure. To index the presence of an intensely pleasurable state, we used the “musical chills” response. Chills, or “shivers-down-the spine”, are well-established physiological phenomena that are experienced in response to music listening [6], [8], [20], [26]–[28]. Musical chills are not experienced by everyone, but those who do experience them tend to do so consistently during moments of peak pleasure. Importantly, chills involve stereotypical changes in physiological arousal [20], [27], [29], [30] that can be objectively indexed to verify their occurrence. Finally, each individual was asked to listen to all other participants' “chills music” selections, and rate how much pleasure they felt as a result of listening to it, from none to intense pleasure. Using this technique, each musical excerpt was matched with two individuals, one who experienced intense pleasure to it, and one who experienced no pleasure to it. This method allowed for a direct comparison of changes in physiological responses to the music, allowing us to isolate any stimulus-driven changes that might be observed in both participants versus response-driven changes that are individualized.

We also needed a reliable indicator of emotional arousal. Traditionally, this has been obtained by asking people to evaluate their emotional state after listening to each excerpt [10], [25], [31]–[33]. However, there are two problems with this method, particularly for the purposes of our study. First, subjective ratings of emotional arousal may be too arbitrary for comparisons across different participants since one person may consider a major change in arousal as a one point increase on the scale, whereas another participant may consider only a minor change sufficient for this increase. To account for this, we implemented a more objective method by recording the physiological signals mediated by the autonomic nervous system that are highly sensitive to emotional arousal, including changes in electrodermal activity, heart rate, blood volume pulse (BVP) amplitude, respiration rate, and body temperature [8], [34]. The second problem is that music is a dynamically changing stimulus. That is, any associated emotions and intensely pleasurable responses (such as those marked by chills) develop over time. Thus, a single value averaged over the entire stimulus would not fully capture the second-by-second changes in subjective and physiological states. To correct for this problem, our paradigm involved continuous real-time millisecond recordings of both pleasure and physiological arousal, which allowed us to capture the dynamic relationships between the two with high temporal sensitivity.

In summary, to overcome previous limitations, simultaneous recording of subjective pleasure states and objective indicators of emotional arousal recorded in real-time were used to capture any systematic relationships between the two. We tested the hypothesis that if the rewarding aspects of music listening are indeed a result of the emotional states produced, there would be a positive correlation between emotional arousal and pleasure states. It further follows that a lack of pleasurable responses should also be accompanied by low emotional arousal.

## Methods

### Participants

Two hundred and seventeen participants responded to advertisements posted around the university campus and sent to various university and community email lists. The advertisement recruited individuals who experience “chills” as an intensely pleasurable response to music. Three rounds of screening were implemented (email, telephone, in-person interview) to assess whether participants met the following criteria: (1) experienced chills consistently to a piece of instrumental music, and (2) chills were pleasurable (i.e., not experienced because the individual was cold, surprised, or frightened) and did not diminish greatly with multiple listening. In addition, the chill-inducing music could not contain any lyrics or be associated with a specific memory (see Stimuli section). Individuals who met all the above criteria were selected for the study. A sample of over 200 chill-inducing music selection submitted through the first round of recruitment can be found in supplementary files online (Table S1). Participants who met all the exclusion criteria and were selected for the study included 17 women and 15 men, between the ages of 18 to 36 (*M* = 22, +/− 3.8 years), with a broad range of musical experience (no training to 18 years of experience). All individuals were healthy and free from any neurological or psychological disorders, assessed through a prescreening interview, and gave written informed consent before participating in the study. Ethical consent for the study was approved by the Montreal Neurological Institute Internal Review Board.

### Stimuli

Participants were instructed to provide 3–5 pieces of intensely pleasurable instrumental music to which they experience chills. Prescreening interview questionnaires were implemented to ensure that chills are experienced consistently each time the participant listens to the music and experienced at moments of intense pleasure, rather than when the participant is surprised or scared. An important exclusion criterion was that the selected music could not be specifically or generally associated with an episodic memory. That is, the music could not be associated with a specific life event (e.g., prom, a memorable concert, etc.) or a period of time (e.g., summer of 2004, high-school, etc.). Movie soundtracks were acceptable as long as the individuals had not seen the movie. These criteria were implemented to decrease extra-musical associations [3], and ensure that the music was pleasurable in itself and not acting as a trigger for emotional memories.

There were no restrictions to the genre of music that could be provided. This was done to increase the ecological validity of our findings and to ensure that any observed effects were not due to a specific genre of music. We obtained music from various genres, including classical, folk, jazz, electronica, rock, punk, techno, and tango (see Table 1 for a sample list of excerpts used in study), with a wide range of psychoacoustical parameters. The modal genre was classical music. Musical clips were cut down to three-minute clips to ensure consistency. Time-frame guidelines to cut the clips were selected by participants to represent the most pleasurable section of each musical excerpt.

**Table 1:** Self-Selected Musical Excerpts.

Title	Composer/ Artist	Genre	Considered Pleasurable	Considered Neutral
Canon in D	Pachelbel	Classical	P1	P10
Clair de Lune	Debussy	Classical	P2	P22
Adagio for Strings	Barber	Classical	P3	P5
Adagio for Strings	Barber	Classical	P4	P17
Requiem – Lacrimosa	Mozart	Classical	P5	P3
Second Symphony	Beethoven	Classical	P6	P24
New World Symphony	Dvorak	Classical	P7	P18
Moonlight Sonata	Beethoven	Classical	P8	P4
Swan Lake	Tchaikovsky	Classical	P9	P1
Romeo and Juliet	Prokofiev	Classical	P10	P6
Piano Concerto 2	Shostakovich	Classical	P11	P14
Fifth Symphony	Shostakovich	Classical	P12	P15
Symphonie Fantastique	Berlioz	Classical	P13	P20
Pines of Rome	Respighi	Classical	P14	P11
Second Symphony	Mahler	Classical	P15	P23
Rhapsody on a Theme of Paganini	Rachmaninoff	Classical	P16	P19
Morceaux de Fantasies	Rachmaninoff	Classical	P17	P26
Elegy	Elgar	Classical	P18	P7
Claressence	Holland	Jazz	P19	P8
Shine on You Crazy Diamond	Pink Floyd	Rock	P20	P21
Nyana	Tiesto	House	P21	P16
Hardstyle Disco	Biomehanika	Trance	P22	P13
Horns of a Rabbit	Do Make Say Think	Post-Rock	P23	P9
Lincolnshire Posy	Grainger	Folk	P24	P25
Jamedaran	Alizadeh	International	P25	P12
Vicious Delicious	Infected Mushroom	Psychedelic Trance	P26	P2

Excerpts used in the study, including title, composer, and genre, are presented in Table 1. To account for psychoacoustical differences between self-selected chill-inducing stimuli, each excerpt was used once as an experimental and once as a control stimulus. In other words, each excerpt was matched with one participant who considered it pleasurable and one who considered it neutral, as indicated in the last two columns.

### Procedures

Participants were tested individually over one session. During the first half of the session, each individual listened to one-minute clips of all musical pieces provided by other participants and rated each piece on a subjectively experienced pleasure scale of 1–10 (1 = neutral, 10 = extremely pleasurable). Musical clips represented the most pleasurable minute of each excerpt, as previously selected by participants, and included the point at which chills are experienced. Participants were then asked to choose the most familiar pieces amongst those that they had rated the lowest on pleasure. This step was taken to decrease differences in familiarity across their self-selected pleasurable and “neutral” music. Individuals often selected pieces that they knew well but found “boring”. If participants were not familiar with any of their low rated pieces, they were played the selections up to two more times to increase familiarity. Thus, the most familiar pieces rated low on pleasure were then selected as control stimuli for each person. Individuals were also asked to identify any musical pieces that were unpleasant. These excerpts were not used as control stimuli because they are not representative of a relatively “neutral” state, but rather may contribute to arousal indicative of annoyance or displeasure.

**Figure 1:**
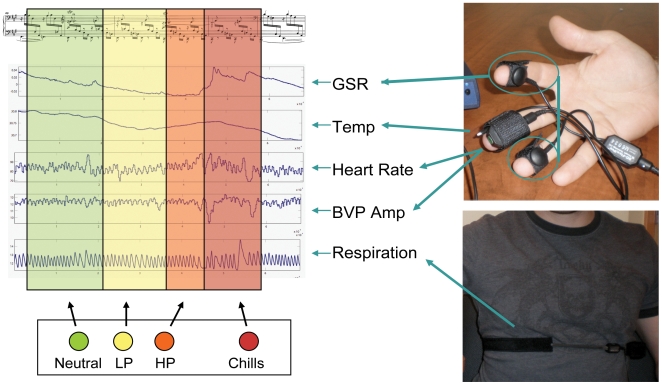
Assessment of Pleasure and Emotional Arousal. Emotional arousal was assessed through psychophysiological measurements of galvanic skin response (GSR), temperature, heart rate, blood volume pulse (BVP) amplitude, and respiration rate. Pleasure states were continuously obtained through subjective ratings of “neutral”, “low pleasure”, and “high pleasure” using a button box. Chills were also indicated through button presses. Psychophysiological correlates of each pleasure state were analyzed to determine systematic relationships between increases in pleasure and emotional arousal.

During the second half of the session, participants were fitted with psychophysiological equipment to record heart rate, BVP amplitude, respiration rate, electrodermal activity or galvanic skin response (GSR), and body temperature. These sensors consisted of 11 mm Ag/AgCl dry electrodes placed on the ring and middle fingers for recording electrodermal activity and secured with Velcro straps, a photoplethysmyograph sensor placed on the middle finger for recording heart rate and BVP amplitude also secured with Velcro straps, and a digital thermometer inserted inside the BVP attachment strap on the index finger for recording peripheral skin surface temperature (Figure 1). A Hall effect respiration sensor was placed around the diaphragm to record respiratory rate. Physiological data were collected with the Procom Infinity biofeedback system by Thought Technology.

When measuring psychophysiological data, it is important to consider baseline differences in physiological activity amongst participants. Since we were interested in *relative* differences in autonomic nervous system activity that can be attributed to music listening, rather than *absolute* differences amongst participants, it would not be valid to directly compare differences amongst participants. Rather, to account for inter-individual differences in physiological activity, recordings during music listening were compared with baseline physiological data that were collected over a 5-minute silent relaxation period for each participant. Subtracting this baseline data provides a difference score indicating the changes in physiological responses that were due to music listening.

All participants were tested in a sound proof laboratory at the Centre for Interdisciplinary Research in Music Media and Technology (CIRMMT). Individuals were asked to position themselves comfortably on a couch in a dimly lit laboratory and all distractions were minimized. Music was played through Sennheiser hd 595 high fidelity headphones and volume was adjusted to a comfortable listening level before beginning the session. After baseline physiological data were collected, six musical excerpts, three “chills” and three “neutral” pieces, were played in a randomized order. While participants were listening to music, they rated the degree of pleasure they were experiencing to the music in real-time (1 = neutral, 2 = low-pleasure, 3 = high-pleasure) using three separate buttons on a game-type input device held in the dominant hand (Figure 1). They were required to hold down the appropriate button as long as they were experiencing the respective degree of pleasure, and press a fourth button when they were experiencing a chill. We tested whether button presses alone could elicit a significant physiological response, which may contaminate the data. Consistent with previous studies, this was not the case [8], [35].

To investigate the degree to which the number and intensity of intensely pleasurable events (marked by chills) contribute to the overall pleasure experienced by a musical excerpt, after hearing each musical selection participants were asked (1) how many chills they experienced; (2) to rate the intensity of each chill (1 = low intensity, 10 = high intensity); and (3) rate the overall degree of pleasure they felt in response to the musical excerpt (1 = not at all pleasurable, 10 = extremely pleasurable). Musical pieces that were still rated as “neutral” on the pleasure dimension (but not described as “annoying” or “unpleasant”) after hearing the entire excerpt were selected to be used for comparison against that same piece played for a participant who rated it extremely high on pleasure. To examine differences between felt and perceived emotions, participants were also asked to rate the valence (1 = sad, 10 = happy) and arousal (1 = not at all aroused, 2 = highly aroused) they felt in response to the musical excerpt, as well as the valence and arousal they believed the composer was intending to convey. This was then followed by another one-minute “rest” condition to ensure that the biosignals have returned to a relaxed resting condition and minimize cross-contamination of psychophysiological effects between subsequent excerpts.

### Data Analysis

During music listening, any changes in subjective evaluations of pleasure states as indicated by participants were recorded continuously. It is important to consider that individuals have different response times; the exact moment at which participants press the button to indicate an increase in pleasure may vary by 1–2 seconds. To avoid miscategorizing pleasurable events, we accounted for reaction time differences between participants by segmenting the data into three-second epochs, during which the highest pleasure rating of that epoch was recorded. Thus, each 3:00 minute clip was divided into 60 three-second epochs, each of which was categorized as “within-excerpt neutral” (WE-neutral), “within-excerpt low pleasure” (WE-LP), “within-excerpt high pleasure” (WE-HP), or “within-excerpt chills” (WE-chills) according to button presses (Fig. 1). In addition, the first two epochs (first six-seconds) of recording for each excerpt was discarded in order to remove data related to any possible startle response associated with music onset.

To prepare physiological data for analysis, signal filtering was performed to remove noise and artifacts. The raw respiration and BVP signals are highly susceptible to noise caused by torso and hand movement, respectively. These artifacts are usually characterized by brief (<100 ms), high frequency and large amplitude spike events or non-linear DC offsets. A third-order Butterworth low-pass filter was convolved with the raw signals to remove the high-frequency contaminants without loss of physiologically-related information. A detrending function using a polynomial regressor was applied to cancel out the DC shifts. The GSR sensor's output exhibits a slow downwards drift over long recording times on some subjects. This is due to charge accumulation at the electrode-skin junction, which causes a linear decrease of conductance. The raw GSR signal was thus detrended using a piecewise linear regression model when this drift was noticed through visual inspection. No filtering was required for the temperature signal as this was outputted clean from the Procom unit.

Despite using individual baseline levels of physiological activity, the data revealed large variability between changes in physiology amongst participants. For example, a participant may have extreme changes in GSR in response to musical stimuli, although this response would remain extreme during all rating conditions (WE-neutral, WE-LP, WE-HP, and WE-chills) and maintain a relative difference between the conditions. As such, if outliers were removed from the data as a group, all data from this participant may be removed, despite their validity. To correct for this problem, outliers beyond four standard deviations from the mean of each rating category (WE-neutral, WE-LP, WE-HP, and WE-chills) were removed for each excerpt and for each participant individually. These outliers are most likely caused by intermittent events unrelated to the music, such as coughing, sneezing, a sudden deep breath, or any other type of a distraction, despite great care taken to minimize distractions as much as possible. Approximately 2–5% of the datapoints were removed for each of the psychophysiological measures: GSR, heart rate, respiration, temperature, and BVP amplitude.

To examine whether within-excerpt changes in psychophysiology are due to individualized emotional reactions to the excerpt or related to the changes in acoustical features of each musical excerpt, we matched each excerpt with one individual who considered it pleasurable and one individual who considered it neutral. Although it was not possible to find a perfect match for all the musical selections, we were able to match 26 excerpts in this way, such that two excerpts were used for each person: one that they considered pleasurable and one that they considered neutral (see Table 1). Thus, the analyses presented here are limited to data collected from these excerpts.

A 2×4 mixed analysis of variance (ANOVA) was performed with Excerpt Type as the repeated measure with two levels (Considered Pleasurable and Considered Neutral), where data were collected on the same excerpt under two conditions, and Within-Excerpt Rating as the second variable with four levels (WE-neutral, WE-LP, WE-HP, and WE-chills). Each psychophysiological measure (GSR, heart rate, respiration, temperature, and BVP amplitude) was separately analyzed as a dependent variable. The values of the Rating condition were determined in the following way: Excerpts were divided into three-second epochs and each epoch was labeled with a rating (i.e., WE-neutral, WE-LP, WE-HP, and WE-chills), based on the button-presses of the individual who found the pieces pleasurable. Once each epoch had an associated Rating condition, the physiological response that corresponded with each epoch was compared between the individual who considered the excerpt pleasurable with the individual who considered it neutral. As such, if physiological responses were significantly different between a WE-LP and a WE-chills epoch, but only for the individual who considered the excerpt pleasurable, we can assume that this increase is really related to increasing pleasure, and not to only to some physical feature of that portion of the excerpt. However, if the increase in physiological response were also observed in the individual who did not consider the excerpt pleasurable, then this change may be due to a specific psychoacoustical parameter of the musical piece (e.g., a sudden increase in tempo). Thus, we examined whether there were significant differences on each psychophysiological measure between the four rating conditions on the excerpts when they were considered pleasurable as compared with when they were considered neutral.

## Results

### Pleasure and Emotional Arousal

**Figure 2:**
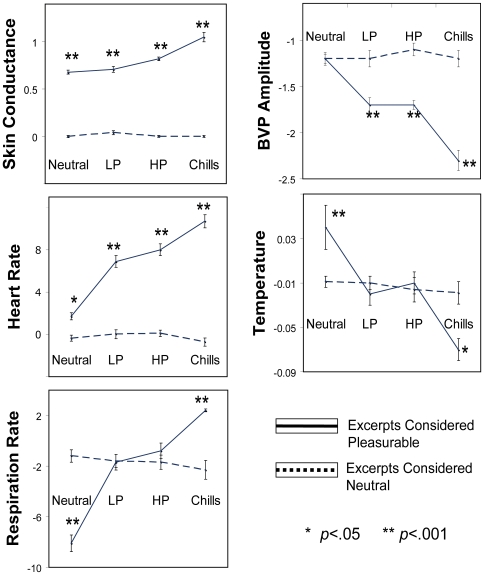
Relationship between Pleasure and Emotional Arousal. Data showed significant positive correlations between subjectively reported pleasure states and objectively measured increases in autonomic nervous system activity for all physiological measures. Participants who reported “no pleasure” on the same epochs of the same musical excerpts revealed no significant changes in physiological activity.

Our data revealed a strong positive association between subjective ratings of pleasure and autonomic nervous system arousal. Figure 2 demonstrates increases in electrodermal activity, heart rate and respiration rate, as well as decreases in temperature and BVP amplitude, as participants report experiencing more pleasure to the musical excerpts; each of these will be discussed individually. Importantly, participants who reported no increases in pleasure in response to the same epochs of the same excerpts did not show any significant increases in autonomic nervous system activity on any of the physiological measures.

#### Electrodermal Activity

The two-way mixed ANOVA revealed a significant main effect of Excerpt Type [F(1,1398) = 2384.32, *p*<.001], indicating that GSR was significantly higher when an excerpt was considered pleasurable than when the same excerpt was considered neutral. More importantly, there was also a main effect of Within-Excerpt Rating [F(3,1398) = 14.99, *p*<.001], providing evidence for physiological changes in GSR *within* excerpts that corresponded to real-time subjective reports of increasing pleasure. Importantly, a significant interaction [F(3,1395) = 20.60, *p*<.001] revealed that within-excerpt changes in GSR were only significant for the Considered Pleasurable group, but not the individual in the Considered Neutral group, suggesting that the changes in GSR were not due to psychoacoustical parameters, but specific to individualized emotional reaction to the same excerpts. For individuals who considered the excerpts pleasurable, GSR increased by 0.072 uS (σ = 0.53) or 10% of its increase from baseline as pleasure ratings progressed from WE-neutral to WE-LP, but this increase was not significant. However, GSR became significantly different from baseline during WE-HP, where it increased by 0.078 uS (σ = 0.55) or 22% (*p*<.01). The largest change was observed during the chills response, where GSR demonstrated a large increase of 0.21 uS (σ = 0.81) or 53% (*p*<.001). There were no significant differences in GSR on the ratings for the Considered Neutral group. Furthermore, post-hoc tests revealed that GSR was higher for the Considered Pleasurable group compared to the Considered Neutral group during all ratings: WE-neutral, WE-LP, WE-HP, and WE-chills (*p*<.001; Figure 2).

#### Heart Rate

Heart rate revealed a highly significant main effect for Excerpt Type [F(1,1371) = 428.32, *p*<.001] and Within-Excerpt Rating [F(3, 1371) = 39.43, *p*<.001], as well as a significant interaction [F(3,1371) = 31.03, *p*<.001]. Thus, similar to GSR, psychoacoustical parameters of the music could not account for changes in HR since only participants in the Considered Pleasurable group demonstrated significant changes. For the Considered Pleasurable group, heart rate increased as participants experienced heightened pleasure states an average of 5.5 beats per minute (BPM; σ = 9.4) or 340% of its increase from baseline as pleasure ratings increased from WE-neutral to WE-LP (*p*<.001). Heart rate continued to increase with increases in pleasure ratings, and although the changes between WE-LP, WE-HP, and WE-chills were not significantly different from each other, they were all significantly different from WE-neutral (*p*<.001). Heart rate increased 6.41 BPM (σ = 11.6) or 398% from WE-neutral to WE-HP, and 8.1 BPM (σ = 8.9) or 500% to WE-chills. There were no significant differences in heart rate on the ratings for the Considered Neutral group, and post-hoc tests confirmed significantly higher heart rate for the Considered Pleasurable group compared to the Considered Neutral group during ratings of WE-neutral (*p*<.01), WE-LP (*p*<.001), WE-HP (*p*<.001), and WE-chills (*p*<.001; Figure 2).

#### Respiration Rate

Respiration rate did not reveal a significant main effect for Excerpt Type, however, there was a significant main effect for Within-Excerpt Rating [F(1,1340) = 23.42, *p*<.001], as well as a significant interaction [F(3,1340) = 28.09, *p*<.001]. For the Considered Pleasure group, respiration rate increased significantly with increases in pleasure ratings. Mean respiration rate increased 6.2 breaths per minute (BtPM; σ = 10.4) or 76% from baseline as pleasure ratings increased from WE-neutral to WE-LP (*p*<.001). Respiration rate increased 7.8 BtPM(σ = 12.9) or 94% from baseline during WE-HP, which was not significantly different from WE-LP, but still a significant increase from WE-neutral. There was another significant increase of 11.18 BtPM (σ = 16.4) or 139% from baseline to WE-chills (*p*<.05). There were no significant differences in respiration on the ratings for the Considered Neutral group. Post-hoc tests revealed that compared to the Considered Neutral group, respiration rates of the Considered Pleasurable group were lower during WE-neutral (*p*<.001*)*, not significantly different during WE-LP and WE-HP, but significantly higher during WE-chills (*p*<.001; Figure 2).

#### Body Temperature

Changes in peripheral skin temperature did not demonstrate a main effect of Excerpt Type, however, there was a significant main effect of Within Excerpt Rating [F(3,1395) = 8.84, *p*<.001], suggesting that changes corresponded with pleasure ratings within-excerpts; and a significant interaction [F(3,1395) = 6.74, *p*<.001], suggesting that within-excerpt changes were not generalizeable to everyone, but only those who found the pieces highly pleasurable. For individuals who experienced pleasure, peripheral skin surface temperature demonstrated a general decrease as participants experienced heightened pleasure states. Temperature decreased 0.087 °C (σ = 0.248) or 138% from baseline as pleasure ratings increased from WE-neutral to WE-LP (*p*<.01), 0.079 °C (σ = 0.184) or 124% to WE-HP (*n.s.*), 0.137 °C (σ = 0.144) or 217% to WE-chills (*p*<.05). There were no significant differences in temperature on the ratings for the Considered Neutral group. Post-hoc tests revealed that compared to the Considered Neutral group, respiration rates of the Considered Pleasurable group were higher during WE-neutral (*p*<.001*)*, not significantly different during WE-LP and WE-HP, but significantly lower during WE-chills (*p*<.05; Figure 2).

#### Blood Volume Pulse Amplitude

Results of the BVP analysis, which reveals peripheral blood vessel vasoconstriction, revealed a significant negative correlation with pleasure ratings. A significant main effect for Excerpt Type [F(1,1392) = 107.66, *p*<.001] and Within-Excerpt Rating [F(3,1392) = 14.70, *p*<.001] were found, as well as a significant interaction [F(3,1392) = 15.38, *p*<.001]. For the Considered Pleasurable group BVP amplitude demonstrated an average decrease of 0.43 (σ = 1.20) or 34% of its decrease from baseline as pleasure ratings increased from WE-neutral to WE-LP (*p*<.01), 0.52 (σ = 1.20) or 41% to WE-HP (*n.s.*), and 1.02 (σ = 1.73) or 81% during WE-chills (*p*<.01). There were no significant differences in temperature on the ratings for the Considered Neutral group, and post-hoc tests confirmed significantly lower BVP amplitude for the Considered Pleasurable group compared to the Considered Neutral group during ratings of WE-LP (*p*<.001), WE-HP (*p*<.001), and WE-chills (*p*<.001; Figure 2).

### The Chills Response

**Figure 3:**
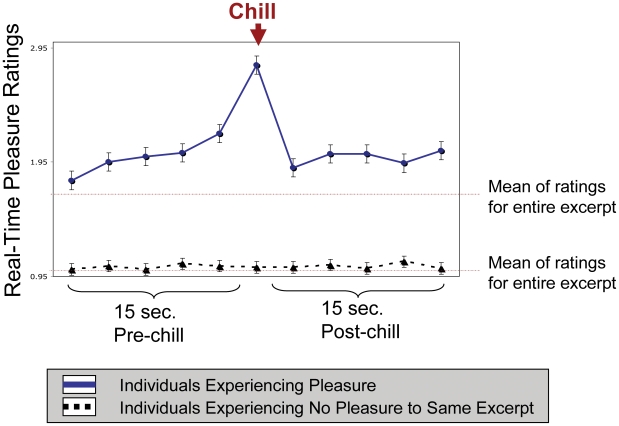
Relationship between Pleasure and the Chills Response. Real-time pleasure ratings plotted against the time-course of the chills response reveal that chills are experienced at the peak of pleasure ratings. Individuals who experienced no pleasure to the same excerpts showed no increases in pleasure during the epochs that chills were experienced in individuals who found the music highly pleasurable.

Data from real-time button presses revealed that out of a total of 310 chills experienced by all participants, 250 were experienced during self-appraised states of highest pleasure (binomial *p*<.0001). In other words, over 80% of chills occurred at the highest moments of pleasure. This confirmed that chills are not random phenomena, but correspond with peak pleasure responses. To examine the nature of the relationship between increases in pleasure ratings and chills onset, ratings were plotted against the time-course of the excerpt. Each excerpt was first divided into 15 second epochs and a one-way ANOVA demonstrated that the only significant change in pleasure ratings involved the 15 second epoch prior to the onset of chills (*F*(10,3780) = 521, *p*<.001; Figure 3). Post-hoc Games-Howell tests revealed that ratings become significantly higher than the mean of ratings for the entire excerpt six seconds before a chill is experienced (mean difference = +.19, *p*<.001), and show another significant increase three seconds before the chill onset (mean difference = +.34, *p*<.001). The peak of the pleasure ratings coincided with the chills response, and decreased significantly thereafter (mean difference = −0.9, *p*<.001). To ensure that the changes in pleasure ratings were due to individualized emotional reactions to the excerpt and not specific to the changes in acoustical features, data for each excerpt was matched with the same piece listened to by a participant who had rated that piece as “neutral”. There were no significant increases in real-time pleasure rating during or prior to the epochs at which chills were experienced for participants who considered the excerpts neutral (Figure 3).

**Figure 4:**
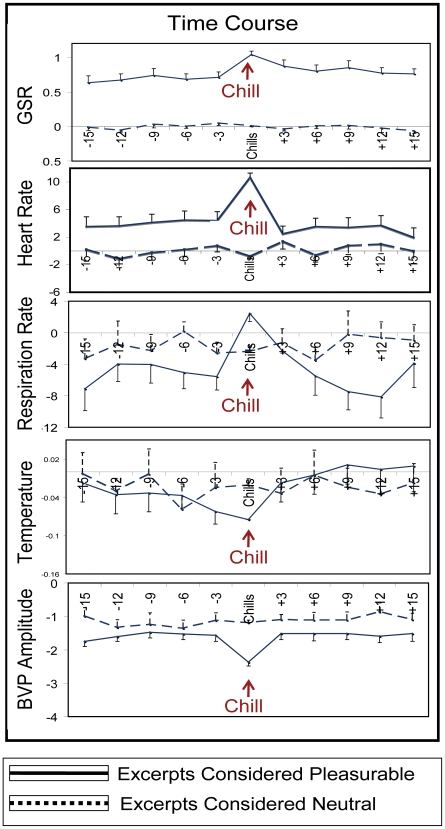
Time-Course of the Chills Response. Real-time physiological recordings plotted against the time-course of the chills response reveal that chills are experienced during the peak of sympathetic nervous system activity. Individuals who experienced no pleasure to the same excerpts did not show significant changes in psychophysiological responses during the epochs that chills were experienced in individuals who found the music highly pleasurable.

To examine the time course of sympathetic nervous system activity leading up to chills, the mean of physiological responses during epochs immediately preceding chills were plotted over time. Figure 4 reveals that all physiological signals tend to show increasing autonomic nervous system arousal during the prechills epochs and peak during the chills response. These trends are not observed in physiological signals of individuals who consider the excerpts neutral, suggesting that they are due to individualized emotional reactions to the excerpt and not specific to changes in acoustical features (Figure 4).

Finally, after listening to each musical excerpt, participants responded to questions about the number and intensity of chills experienced, and rated the degree of pleasure they experienced in response to the entire excerpt. These results revealed that the reported intensity of the chills was highly correlated with the overall pleasure experienced in response to a piece of music (*r* = .74, *p*<.001) and the number of chills experienced during a music excerpt was moderately correlated with this measure (*r* = .29, *p*<.001).

### The Relationship between the Felt and Perceived Emotional Responses

After listening to each musical excerpt, participants rated the degree of valence and arousal they (1) *felt* in response to the excerpt, and (2) believed the excerpt was intended to convey. To investigate the hypothesis that individuals do not always feel the emotions that an excerpt is intended to convey, we analyzed differences between these two ratings. Paired-sampled t-tests confirmed significant differences between the emotions that participants felt in response to an excerpt with that which they believed the music was intended to convey, with respect to both valence (t(235) = 2.46, *p*<.01), and arousal (t(235) = 11.96, *p*<.001).

We had further hypothesized that emotions would be more genuinely felt in response to some musical excerpts, namely the self-selected pieces, more so than experimenter-selected pieces. Thus, we examined self-selected and experimenter-selected musical excerpts separately. Experimenter-selected pieces were those that were not provided by the participant, but brought in by other participants as their self-selected pieces. As such, even the experimenter-selected pieces were considered high on emotionality by some individuals. A two-way fixed-factorial ANOVA was conducted with self-selected versus experimenter-selected as one factor and felt versus perceived as the other. Results revealed a significant interaction for both valence and arousal [F(1, 232) = 7.51, *p*<.01; F(1, 232) = 27.15, *p*<.001), respectively], suggesting that similarities between felt and perceived ratings depend on whether excerpts are self-selected or experimenter selected.

**Figure 5:**
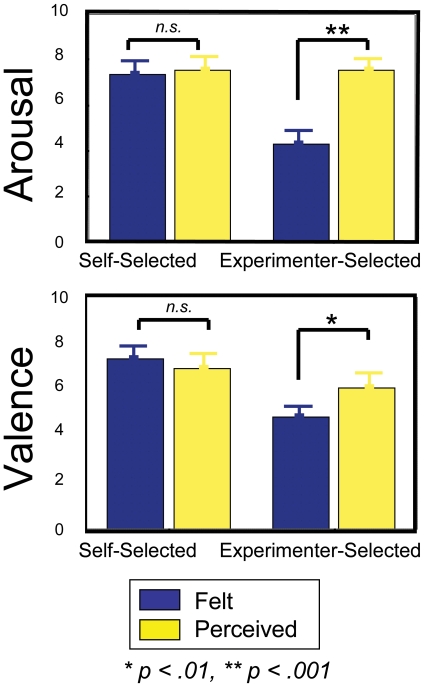
Perceived versus Felt Emotions. Post-listening ratings of valence and arousal revealed significant differences amongst the self-selected and experimenter-selected musical pieces. Self-selected excerpts revealed higher correlations between perceived and felt valence and arousal than experimenter-selected pieces. Furthermore, participants generally reported lower arousal and a more “neutral” valence on experimenter-selected pieces than self-selected pieces.

For self-selected pieces, there were no significant differences between the valence (i.e., degree of happiness or sadness) that participants thought a piece of music was intended to convey, compared to what they felt in response to listening to that excerpt (*M* = 2.28 versus *M* = 1.87 deviation from “neutral”, respectively; S.E. = .30, .29, respectively; Figure 5). Rather, there was a positive correlation between felt and perceived valence with self-selected music (*r* = 0.52, *p*<.001) showing that the two were highly associated. However, for the experimenter-selected pieces, significant differences existed between perceived and felt valence (*M* = 0.21 versus *M* = 1.03 deviation from “neutral”, respectively; S.E. = .23, .33, respectively; Figure 5). In other words, there was no significant correlation between the emotion that the participant felt and what they believed the music was intended to convey (*r* = 0.11, *n.s.*). Additionally, with experimenter-selected music, participants felt more neutral than what they thought the excerpt was intended to convey, despite whether it was a happy or a sad piece. For these excerpts, participants also reported significantly less felt arousal than what they perceived in the excerpts (*M* = 5.02, S.E. = .23 versus *M* = 7.37, S.E. = .19, respectively; Figure 5). Interestingly, the degree of pleasure experienced from an excerpt was most strongly correlated with felt arousal (*r* = .53, *p*<.001), followed by felt valence (*r* = .39, *p*<.001). Importantly, perceived valence demonstrated no significant relationships (*r* = −.043, *n.s.*) with subjective pleasure ratings, suggesting that participants experienced pleasure from excerpts that they believed was intended to convey sadness just as much as that which was intended to convey happiness.

## Discussion

The results of this experiment provide clear evidence for a direct link between emotions and the rewarding aspects of music listening by demonstrating a robust dynamic relationship between increases in emotional arousal and reported increases in pleasure. These findings are also novel insofar as they demonstrate dissociation between physiological responses of individuals who find a given piece of music pleasurable from those who do not, solidifying the predicted connection between emotional arousal and pleasure. Participants who did not report pleasurable states in response to a particular excerpt did not show any significant changes in psychophysiological arousal, whereas others who found those same excerpts pleasurable showed a distinct profile of increasing sympathetic nervous system activity as pleasure increased. These findings provide strong support for the theory that musical emotions underlie the pleasurable aspects of music listening.

Amongst the measures used in the study, electrodermal activity (EDA) is generally considered to be the best predictor of emotional arousal, as it is not under voluntary control, is highly sensitive to autonomic nervous system changes, and is widely used by music and emotion researchers [8], [10], [12], [20], [30], [36]. This measure revealed the best correlation with increases in pleasure ratings, demonstrating a robust increase as subjective reports progressed from low-pleasure to high-pleasure to chills. Even at the lowest pleasure ratings (WE-neutral), participants demonstrated significantly higher EDA on excerpts that they considered pleasurable versus those that were considered neutral. In other words, even listening to the least-pleasurable moments of a musical piece that an individual likes leads to higher EDA than listening to an entire excerpt that the individual finds neutral.

As within-excerpt ratings progressed from neutral to low pleasure, we observed significant increases in heart rate and respiration, and decreases in temperature and BVP amplitude, but only for those who considered the music pleasurable. Interestingly, although all WE-HP states were significantly different from WE-neutral, most measures did not distinguish between WE-LP and WE-HP. One reason for this might be that these measures may not be as sensitive to smaller changes in autonomic nervous system arousal as EDA. However, it is of particular relevance to note that significant differences in mean respiration, temperature, and BVP amplitude were observed during the chills response, not only in relation to WE-neutral, but also in relation to WE-LP and WE-HP. Furthermore, there were significant differences in these variables when comparing across excerpts as well (Fig. 4). This suggests that even the potentially less sensitive measures reveal significant changes during the chills response, alluding to the potency of its affects on the nervous system. The lack of main effect on respiration may be due to the likelihood that rapid respiration rates are often preceded by compensatory periods of deeper and slower breathing. In this case, mean respiration rate for the entire excerpt would not be expected to be very different between the two different excerpt types, but changes in respiration *within* the excerpts would be expected to differ; this was indeed the case.

It is important to clarify that our findings are not intended to suggest that all increases of sympathetic nervous system activity will necessarily lead to increasing pleasurable states. There are other highly aroused states that are not pleasurable (e.g., fear or anxiety). However, these responses can be ruled out in our study, as we focused exclusively on pleasurable stimuli; music clips that aroused unpleasant feelings were omitted as potential stimuli as they do not relate directly to our hypothesis. Thus, the link between pleasure and emotions is unidirectional: an increase in emotional arousal does not necessarily imply pleasurable states, however, pleasurable states are accompanied by emotional arousal.

A second clarification involves the differential contributions of bottom-up and top-down processes to physiological indicators of emotional arousal. Etzel and colleagues (2006) have found that physiological responses, particularly respiration, synchronize with the tempo of a musical excerpt after listening for some time, referred to as tempo entrainment. This process suggests a bottom-up process is leading to changes in physiological activity. However, our results show that two people can have strikingly different physiological responses to a given musical excerpt depending on how pleasurable they find this piece, suggesting a top-down process. Although these findings seem to contradict each other, this is not necessarily the case. There may have been tempo entrainment in our study as well; however, this was accounted for by presenting the same excerpts to both groups. As such, tempo entrainment would be experienced by all and relatively tonic throughout the excerpt. The phasic increases in physiological activity that are more representative of emotional arousal were not observed in all listeners, but only those who reported increases in pleasure, suggesting that they are over and above tempo entrainment.

In a similar vein, other researchers have found that specific psychoacoustical events in music can lead to changes in subjectively reported emotional arousal and psychophysiological indicators of this arousal [28], [30], [37], emphasizing the stimulus-driven properties of musical stimuli in inducing temporary physiological changes. We took this into account by defining events based on subjective ratings of those who found the music pleasurable, which do not necessarily coincide with changes in psychoacoustical parameters. In other words, a sudden change in loudness or an unexpected harmony may lead to transient stimulus-driven changes in psychophysiology across all listeners, but these nuances will be masked since the epochs during which those who experienced pleasure enjoyed the music are relatively longer and may not necessarily contain these psychoacoustical features. Thus, although it is quite likely that specific psychoacoustical parameters resulted in some psychophysiological changes, these would be expected across all participants, and the differential autonomic activation that was observed across groups is over and above exclusively bottom-up processes. It is likely that a combination of highly individualized top-down processes (e.g., expectancies) that are tightly connected to the pleasurable aspects of a given piece of music [5] and bottom-up processes (e.g., fulfillment of those expectancies) interact to result in the intense sympathetic nervous system arousal observed over and above exclusively bottom-up processes that are experienced similarly for everyone.

Numerous studies have examined the autonomic activation patterns elicited by different musical emotions, such as happy sad, and fearful [10], [12], [13], [25], [33], [38]–[45]. However, a number of these studies have found inconsistent results (see [32], [46] for a review), and specific patterns of psychophysiological activity have not been established for the experience of different emotions induced by music. These inconsistencies challenge the emotivist perspective, as one would expect greater consistency in the experience of musical emotions if they were experienced in a similar manner to basic emotions. Our results provide some clarification for the reasons behind the inconsistencies. The first points to a methodological concern involving the difference between felt and perceived emotions (also see [7], [28]). Our findings demonstrate that the emotions felt in response to a musical piece are not necessarily the same as those that the music is intended to convey. In our study, participants were asked to rate separately the emotion and degree of arousal that they *perceived* in the music (i.e., thought it was intended to convey), with that which they *felt* in response to listening to it. The results demonstrated highly significant differences, suggesting that individuals can acknowledge that a piece of music may intend to invoke a particular emotion that is different from the emotion that they are feeling. For example, it was not uncommon for people to rate an excerpt high on valence and arousal when asked “What do you think this excerpt is intended to convey?”, but also low on valence and arousal when asked “How did you feel when listening to this excerpt?”. This finding reveals a major flaw in current paradigms of research with music and emotions, with compulsory implications for future studies. Although it seems intuitive that felt emotions are different than perceived emotions, this distinction has not been acknowledged in the majority of previous studies (see [47]). If perceived and felt emotions are not explicitly differentiated for subjects in a study, they may be assumed to be similar by that individual, and lead to a demand characteristic (e.g., “This is a happy piece of music, therefore, I should say that I'm happy.”). However, what is essentially being examined in these situations will involve emotion *recognition*, rather than the subjective experience of the emotion, which may explain some of the inconsistencies in findings. A second reason for inconsistencies is the finding that an individual may experience pleasure to music that is intended to convey sadness. Although this seems counterintuitive, our results demonstrate that intense pleasure can be experienced in response to both happy and sad music, but importantly, the degree of pleasure experienced in response to the music mediates emotional arousal, regardless of whether happiness or sadness was conveyed by the music. Thus, these findings suggest that identifying specific patterns of psychophysiological activity associated with musical emotions is complicated by highly individualized top-down processes involving the degree of pleasure experienced in response to a piece of music. Further, since it has been well-established (and further confirmed in our study) that musical preferences vary widely, it is likely that inconsistencies in previous studies are arising from more complex phenomenon relating to interpersonal differences. Musical emotions are unique from other emotions in that they are based on aesthetic stimuli, and unlike other basic emotions do not have an obvious adaptive or survival value [48]. For this reason, it would be expected that the experience of them would not be universally similar, but highly individualized depending on personal preferences and previous experiences. Our data support this hypothesis, and provide new avenues for future research. More specifically, it remains to be determined how interpersonal factors, such as personal preferences, familiarity, and expectations contribute to emotional responses to musical stimuli (also see [5]).

One might argue that factors such as familiarity or associations may act as confounding variables when using self-selected musical stimuli. That is, it may be difficult to parse out the extent to which the observed results are due to differences between listening to familiar versus novel music. We accounted for this issue in several ways. First, we made several attempts to decrease any differences in degree of familiarity between pleasurable and neutral excerpts. After participants selected the excerpts to which they felt most “neutral”, they were asked to choose amongst those that they were most familiar with; these pieces were then selected as that participant's control. This was possible as many of the pieces were popular and well-known classical pieces. In the case that participants were not sufficiently familiar with any of their selected “neutral” excerpts, the pieces were replayed for them two more times to increase familiarity. Thus, for the most part, we tried to minimize differences in familiarity across conditions as much as possible. Although broadly speaking, music that is familiar can be more pleasing, this is not always the case. For example, many familiar pieces of music may be considered “boring”. Similarly, some pieces that may be pleasing at some point become less so with repetition (e.g., TV commercials, popular songs, etc.). This was indeed the case in our study where some participants reported selecting a familiar piece as “neutral” because they were “bored” with it (e.g., Pachelbel's Canon, Barber's Adagio for Strings, Moonlight Sonata, Clair de Lune, etc.). Since familiarity with a musical excerpt is a subjective phenomenon, it would be unreasonable for us to assume that we can control for it entirely, unless all the selections were novel. However, as another way to deal with the issue we also took advantage of the *within*-excerpt design. It can be safely assumed that participants are familiar with an entire piece of music, and not just the brief parts that they find most highly pleasurable. Since the results showed significant changes in psychophysiological responses within the excerpts, and these correlated with self-reported increases in pleasure, we can rule out the possibility that changes in psychophysiological responses were entirely due to familiarity rather than increases in pleasure.

A second related confounding variable involves associations of music with autobiographical memories. It is difficult to assess the extent to which music may be triggering pleasurable memories, and it is likely impossible to rule out all associations to memory with self-selected stimuli, as some may be unconscious. However, we took steps to rule out any obvious associations with memory. For this reason, participants were asked if the music was associated with (1) a specific memory (e.g., prom, a memorable concert, etc.), or (2) a period of their lives (e.g., summer of 2004, high-school, etc.). This was indeed the case for a number of individuals amongst the initial pool of 217 potential participants, who were then excluded from participating in the study. Once again, however, it is important to note the *within*-excerpt changes in physiology in response to the pleasurable pieces, which decrease the possibility that the effects were entirely due to recalling autobiographical events, since these would be relatively consistent throughout the piece.

The findings of this study can be differentiated into two main categories: those that involve increases in pleasure, and those that are specifically associated with the chills response. The latter can only be generalized to individuals who do experience chills to music. Although this limits the generalizeability of this part of the findings, one purpose of the study was to specifically examine these intense events. The first category relating to pleasure, however, can be generalized more widely. Those results showed that even without the experience of chills, self-reported increases in pleasure (subjective) are directly associated with increases in psychophysiological arousal (objective). This point is best demonstrated by the finding that participants often experienced “high pleasure” that did not give them chills, but nonetheless demonstrated significant increases in psychophysiology during these periods. This suggests that physiological arousal and pleasure show a direct correspondence when assessed in “real-time”, even without the experience of chills. The chills phenomenon is of interest because when they are experienced, there are extreme increases in autonomic nervous system arousal that are much more significant that those relating to “high pleasure”. Previous studies had demonstrated that chills are subjectively described as pleasurable [6], [20], [27], and their occurrence generally corresponds with increases in heart rate and electrodermal activity [8], [20], [29], [30]. Our study corroborated these findings with methods involving higher temporal resolution, which allowed us to examine the time-course of physiological activity leading up to the chills response. Results revealed that chills represent peak autonomic nervous system arousal, demonstrating extreme sympathetic nervous system activity. Physiological measures of heart rate, respiration rate, electrodermal activity, skin temperature, and BVP amplitude display a gradual change preceding the chills response and highly significant arousal was observed immediately prior to, and during, the chills response. Subjectively, the chills response is preceded by increases in pleasure ratings that reach a maximum when chills are experienced, and decreases significantly post-chills, further confirming that chills are experienced at the climax of the pleasurable responses. It may be the case that chills are a physiological byproduct of a sudden or intense increase in autonomic nervous system activity. These findings provide strong evidence for Blood and Zatorre's (2001) hypothesis that the chills response is a physical manifestation of the most rewarding experiences to music listening. Additional support comes from our finding that the number and intensity of chills contribute significantly to the overall pleasure experienced from the musical excerpt, with the latter being a stronger predictor. This is consistent with Blood and Zatorre's results that the intensity of the chills ratings is positively correlated with the degree of blood flow to the ventral striatum, an area implicated in processing pleasurable experiences in response to reward [49]–[51]. These results provide evidence that individuals can experience intensely emotional and rewarding responses to self-selected music, which in turn can lead to chills that act as distinct physiological markers of extreme autonomic nervous system arousal experienced during the climax of pleasure responses to music. It is also of particular significance that Blood and Zatorre (2001), found neural activity in regions of the brain typically involved in emotional processing, namely the amygdala, insula, orbitofrontal cortex, and ventral medial prefrontal cortex while participants were listening to chills-inducing music. This finding further supports the link between emotion and pleasure. The causal link between emotional arousal and reward has long been speculated [52], [53] but difficult to test empirically, since pleasure is a highly subjective phenomena.

In summary, the results of our study provide clear evidence for a relationship between pleasure and emotional arousal. It may be argued that individuals often enjoy music that “relaxes” them, which seems contradictory to our results. However, although moderate enjoyment of music is not necessarily accompanied by major changes in emotional arousal, as pleasure intensifies so do physiological signs of emotional arousal. Individuals listen to music for a variety of reasons, and a wide spectrum of changes in emotional states or pleasurable responses may be experienced in response to music. At the lower end of the pleasure spectrum are mild changes in mood, and at the highest end of the spectrum are intensely rewarding experiences, such as those that induce chills as a physiological response. The latter is of particular significance since such intense pleasure states are rarely caused by stimuli that have no pragmatic, instrumental, or apparent survival value. The intensity of pleasure experienced from music listening has lead some researchers to suggest that it may act upon the dopamine reward system of the brain [54], which is implicated in processing highly rewarding stimuli such as cocaine and amphetamines [55], [56], food [57] , and playing videogames [49]. The assumption that music may also involve this system is largely based on brain imaging findings that have found increasing blood flow or oxygenation to striatal regions of the brain that are implicated in reward, particularly the ventral striatum, or nucleus accumbens [27], [58]–[60]. These imaging studies have also found neural activity in surrounding limbic regions, indicative of emotional arousal. Whether or not dopamine is actually involved remains to be determined, but the present findings suggest that musical pleasure is associated with physiological markers which are consistent with the experience of reward.

Assessment of emotional responses to music, particularly the ability for music to induce highly pleasurable feelings, has become a topic of interest to music researchers with practical implications for music composition, therapy, and marketing. The present data provide a direct link between emotions and pleasure in music listening, and reveal new avenues for research to examine whether strongly felt emotions can be rewarding in themselves in the absence of a physically tangible reward or a specific functional goal.

## Supporting Information

Table S1 Sample of Chills-Inducing Musical Excerpts. This table lists some examples of chills-inducing musical excerpts provided by participants who were initially recruited for the study. Many of these excerpts were not used because the clips or the participants who submitted them did not meet all the exclusion criteria for the study, as outlined in the Procedures section. The times at which individuals experienced chills to the excerpts are listed, however, these times are likely to vary depending on which recording is used.
(0.38 MB DOC)
