# Supplementary material for: The Rewarding Aspects of Music Listening Are Related to Degree of Emotional Arousal
Source: PLoS One. 2009 Oct 16;4(10):e7487. doi: 10.1371/journal.pone.0007487 (PMC2759002; doi:10.1371/journal.pone.0007487)
Supplement: Table S1 — Sample of Chills-Inducing Musical Excerpts. This table lists some examples of chills-inducing musical excerpts provided by participants who were initially recruited for the study. Many of these excerpts were not used because the clips or the participants who submitted them did not meet all the exclusion criteria for the study, as outlined in the Procedures section. The times at which individuals experienced chills to the excerpts are listed, however, these times are likely to vary depending on which recording is used. (0.38 MB DOC) [file pone.0007487.s001.doc]

| **Composer/Artist** | **Title** | **Genre** | **Chills** |  |  |
| --- | --- | --- | --- | --- | --- |
|  |  |  |  |  |  |
| Dave Matthews Band | #34 | Alternative Rock | 1:40 |  |  |
| Everclear | El Distorto Melodico | Alternative Rock | 0:30 | 1:20 |  |
| The Dissociatives | Paris Circa 2007 Slash 08 | Alternative Rock - Experimental | 1:30 |  |  |
| Rutter | What Sweeter music – Choir and Orchestra | Choir | 1:57 |  |  |
| Sheremetiev | Rejoice Now Heavely Powers – Men’s Russian Choir | Choir | 3:30 |  |  |
| Bach | Brandenburg Concerto No. 5 | Classical | 4:18 |  |  |
| Bach | Cello Suite 1 - Prelude | Classical | 2:29 |  |  |
| Barber | Adagio for Strings | Classical | 6:30 |  |  |
| Barber | Adagio for Strings | Classical | 0:25 | 3:40 | 5:40 |
| Barber | Adagio for Strings | Classical | 5:00 |  |  |
| Bartok | Rhapsody No. 1 - Mov. 1 | Classical | 3:00 |  |  |
| Beethoven | 5th Piano Concerto - Mov. 2 | Classical | 0:02 |  |  |
| Beethoven | 5th Symphony - Mov 2 | Classical | 0:16 | 2:02 | 2:36 |
| Beethoven | 9th Symphony - Mov. 2 | Classical | 0:29 |  |  |
| Beethoven | 9th Symphony - Mov. 2 | Classical | 0:30 |  |  |
| Beethoven | Piano Sonata No. 14 in C#-Minor ("Moonlight") | Classical | 1:15 | 3:00 |  |
| Beethoven | Piano Sonata No. 14 in C#-Minor ("Moonlight") | Classical | 3:10 | 4:08 |  |
| Beethoven | Piano Sonata No. 17 in D Minor ("The Tempest") | Classical | 5:33 |  |  |
| Beethoven | Sonata No. 8 in C Minor - Rondo Allegro | Classical | 0:15 | 1:29 | 1:39 |
| Beethoven | String Quartet in B Flat Op. 130 (Cavatina) | Classical | 1:00 |  |  |
| Beethoven | Symphony No. 7 in A Minor - Mov. 2 | Classical | 1:00 |  |  |
| Beethoven | Violin Sonata, No. 5 “Spring" - Mov. 1 | Classical | 0:30 | 1:38 | 2:20 |
| Berlioz | Le Carnaval Romain, Op. 9 | Classical | 8:32 |  |  |
| Berlioz | Symphonie Fantastique, Op. 14 - Mov. 4 | Classical | 2:55 |  |  |
| Brahms | String Quartet No. 1 - Mov. 2 | Classical | 0:39 | 0:47 | 1:12 |
| Brahms | Symphony 4 in E Minor, Op. 98 - Mov. 4 | Classical | 4:23 |  |  |
| Buxtehude | Toccata in G | Classical | 0:40 |  |  |
| Chopin | Ballade No. 1 in G Minor | Classical | 2:03 |  |  |
| Chopin | Mazurka in A Minor Op. 17 | Classical | 0:59 | 1:57 |  |
| Chopin | Nocture in G Minor | Classical | 1:20 | 2:20 |  |
| Chopin | Prelude No. 4 in E Minor | Classical | 0:40 | 1:05 |  |
| Copland | Appalachian Spring Suite | Classical | 20:00 |  |  |
| Debussy | Clair de Lune | Classical | 2:03 |  |  |
| Debussy | Clair de Lune | Classical | 4:30 |  |  |
| Debussy | Clair de Lune | Classical | 1:26 | 2:09 |  |
| Debussy | Images - i-Lent | Classical | 1:00 |  |  |
| Debussy | Prelude to the Afternoon of a Faun | Classical | 4:30 |  |  |
| Dvorak | New World Symphony - Mov. 2 | Classical | 2:30 |  |  |
| Dvorak | New World Symphony - Mov. 2 | Classical | 1:36 | 2:12 |  |
| Dvorak | New World Symphony - Mov. 4 | Classical | 0:20 |  |  |
| Dvorak | New World Symphony - Mov. 4 | Classical | 5:30 | 8:30 |  |
| Dvorak | New World Symphony - Mov. 4 | Classical | 8:30 |  |  |
| Dvorak | Symphony No. 8 - Mov. 1 | Classical | 6:45 |  |  |
| Dvorak | Symphony No. 8 - Mov. 4 | Classical | 2:15 | 9:00 |  |
| Elgar | Cello Concerto - Mov. 1 | Classical | 2:53 |  |  |
| Elgar | Cello Concerto - Mov. 1 | Classical | 1:15 | 4:15 |  |
| Elgar | Elegy | Classical | 0:08 |  |  |
| Fauré | Violin Sonata in A Major - Mov. 1 | Classical | 0:24 | 0:31 | 0:36 |
| Handel | Concerti Grossi, Op. 3, Concerto 2 - Allegro | Classical | 1:10 |  |  |
| Handel | Concerti Grossi, Op. 6, Concerto 4 - Allegro | Classical | 2:25 |  |  |
| Haydn | Concerto in C Major - Adagio | Classical | 1:42 |  |  |
| Haydn | Sonata No. 58 - Mov. 1 | Classical | 0:22 |  |  |
| Holst | First Suite in E Flat | Classical | 9:07 | 9:50 |  |
| Holst | The Planets - Jupiter | Classical | 4:48 | 5:10 |  |
| Holst | The Planets - Jupiter | Classical | 3:05 |  |  |
| Holst | The Planets - Venus | Classical | 4:27 | 4:39 |  |
| Kreisler | Praeludium and Allegro | Classical | 2:20 | 4:30 |  |
| Liszt | Danse Macabre | Classical | 0:27 | 3:37 | 4:21 |
| Mahler | Symphony No. 1 - Mov. 1 | Classical | 14:00 |  |  |
| Mahler | Symphony No. 1 - Mov. 4 | Classical | 5:42 | 9:57 | 15:15 |
| Mahler | Symphony No. 2 - Mov. 1 | Classical | 1:47 |  |  |
| Mahler | Symphony No. 2 - Mov. 1 | Classical | 13:00 | 10:20 |  |
| Mahler | Symphony No. 2 - Mov. 3 | Classical | 7:59 |  |  |
| Mahler | Symphony No. 2 - Mov. 5 | Classical | 7:45 | 15:29 | 21:57 |
| Mahler | Symphony No. 5 - Mov. 1 | Classical | 0:05 |  |  |
| Mozart | A Little Night Music | Classical | 0:17 |  |  |
| Mozart | Requiem: Lacrimosa | Classical | 0:46 |  |  |
| Mozart | Requiem: Lacrimosa | Classical | 0:50 |  |  |
| Mussorgksy | Pictures at Exhibition | Classical | 5:16 |  |  |
| Pachelbel | Canon in D Major | Classical | 2:02 |  |  |
| Penderecki | Threnody for the Victims of Hiroshima | Classical | 0:20 | 3:00 |  |
| Prokofiev | Romeo and Juliet Suite No. 2: Juliet the Young Girl | Classical | 0:20 |  |  |
| Prokofiev | Romeo and Juliet Suite No. 3: Juliet’s Death | Classical | 4:30 |  |  |
| Rachmaninoff | Morceaux de Fantaisie, No. 2, Prelude in C# Minor | Classical | 2:20 |  |  |
| Rachmaninoff | Morceaux de Fantaisie, No. 2, Prelude in C# Minor | Classical | 1:31 |  |  |
| Rachmaninoff | Piano Concerto in C Minor, No. 2 - Mov. 2 | Classical | 1:32 | 8:48 |  |
| Rachmaninoff | Piano Concerto No. 3 | Classical | 1:03 |  |  |
| Rachmaninoff | Prelude No. 4 - D Major | Classical | 0:09 |  |  |
| Rachmaninoff | Rhapsody on a Theme of Paganini | Classical | 1:25 | 2:30 |  |
| Ravel | Gaspard de la Nuit: Ondine | Classical | 0:50 | 3:13 | 3:41 |
| Ravel | Oiseaux Tristes | Classical | 0:23 | 1:04 | 2:05 |
| Ravel | String Quartet - Mov. 2 | Classical | 0:02 | 0:24 | 3:16 |
| Respighi | Pines of Rome | Classical | 6:48 |  |  |
| Rimsky-Korsakov | Scheherazade - Mov. 2 | Classical | 2:50 | 3:17 |  |
| Rimsky-Korsakov | Scheherazade - Mov. 3 | Classical | 6:13 |  |  |
| Saint-Saens | Symphony No. 3 - Mov. 1 | Classical | 1:00 | 3:00 |  |
| Schumann | Fantasy in C - Mov. 1 | Classical | 7:55 |  |  |
| Shostakovich | 11th Symphony - Mov. 2 | Classical | 13:45 |  |  |
| Shostakovich | Piano Concerto No. 2 - Mov. 2 | Classical | 6:45 |  |  |
| Shostakovich | Symphony No. 10 - Mov. 2 | Classical | 1:00 | 2:20 |  |
| Shostakovich | Symphony No. 11 - Mov. 2 | Classical | 14:00 |  |  |
| Shostakovich | Symphony No. 11 - Mov. 4 | Classical | 10:00 |  |  |
| Shostakovich | Symphony No. 4 - Mov. 3 | Classical | 20:30 | 22:30 |  |
| Shostakovich | Symphony No. 5 - Mov. 3 | Classical | 1:08 |  |  |
| Strauss | Voices of Spring Waltz | Classical | 5:20 |  |  |
| Stravinsky | Firebird Suite | Classical | 3:01 | 7:13 |  |
| Stravinsky | Rite of Spring - Augurs of Spring | Classical | 1:45 |  |  |
| Stravinsky | Rite of Spring - The Adoration of the Earth | Classical | 8:45 |  |  |
| Tchaikovsky | Dance of the Sugar Plum Fairy | Classical | 1:15 | 2:00 |  |
| Tchaikovsky | Dance of the Sugar Plum Fairy | Classical | 0:09 |  |  |
| Tchaikovsky | Symphony No. 4 - Mov. 1 | Classical | 4:00 | 8:00 | 17:13 |
| Tchaikovsky | Symphony No. 4 - Mov. 1 | Classical | 12:32 |  |  |
| Tchaikovsky | Symphony No. 5 - Mov. 1 | Classical | 0:00 | 2:50 | 8:39 |
| Tchaikovsky | Symphony No. 5 - Mov. 4 | Classical | 7:16 |  |  |
| Tchaikovsky | Violin Concerto in D - Mov. 1 | Classical | 0:52 | 1:10 | 1:22 |
| Tchaikovsky | Waltz of the Snowflakes | Classical | 1:53 | 3:45 | 5:30 |
| Vivaldi | Four Seasons - Spring | Classical | 0:15 |  |  |
| Vivaldi | Four Seasons - Spring | Classical | 0:16 |  |  |
| Vivaldi | Four Seasons - Summer | Classical | 0:49 | 1:05 | 1:21 |
| Vivaldi | Four Seasons - Winter | Classical | 0:40 | 1:15 | 1:37 |
| Vivaldi | Four Seasons - Winter | Classical | 1:15 | 2:10 | 3:00 |
| Wagner | Lohengrin Prelude to act 1 | Classical | 6:22 |  |  |
| Wagner | Overture to Tannhauser | Classical | 1:00 |  |  |
| Walton | Violin Concerto - Mov. 1 | Classical | 0:30 | 0:59 | 1:16 |
| Echoes | Digitalism | Electronic | 0:31 |  |  |
| Lamb | Angelica | Electronic | 0:05 | 0:27 |  |
| Jimmy Swift Band | 80's Runaway Model | Electronic Rock | 3:07 |  |  |
| Yann Tiersen | Le Moulin | Film Score (Amelie Poulin) | 2:10 |  |  |
| Yann Tiersen | Rue des Cascades - C'etait Ici | Film Score (Amelie Poulin) | 1:13 |  |  |
| Wendy Carlos | Symphony No. 9 - Mov. 2 | Film Score (Clockwork Orange) | 2:15 |  |  |
| Alexandre Desplat | By the Canal with Pieter | Film Score (Girl with a Pearl Earring) | 0:04 |  |  |
| Desplat | Griet’s Theme | Film Score (Girl with a Pearl Earring) | 0:06 |  |  |
| Hans Zimmer | Am I Not Merciful? | Film Score (Gladiator) | 4:48 |  |  |
| Jon Brion | Later Monday | Film Score (I Heart Huckabees) | 1:00 |  |  |
| Ennio Morricone | L'Arena | Film Score (Kill Bill) | 1:15 | 2:00 | 3:05 |
| Shore | A Journey in the Dark | Film Score (Lord of the Rings) | 2:50 |  |  |
| Shore | Amon Hen | Film Score (Lord of the Rings) | 1:05 |  |  |
| Shore | Concerning Hobbits | Film Score (Lord of the Rings) | 0:30 | 1:20 |  |
| Shore | Riders of Rohan | Film Score (Lord of the Rings) | 2:20 |  |  |
| Shore | Samwise the Brave | Film Score (Lord of the Rings) | 0:40 |  |  |
| Shore | The Breaking of the Fellowship | Film Score (Lord of the Rings) | 0:10 | 0:55 |  |
| Shore | The Uruk-Hai | Film Score (Lord of the Rings) | 1:30 |  |  |
| La Caution | The a la Menthe | Film Score (Ocean's Twelve) | 1:20 |  |  |
| Ennio Morricone | The Man with the Harmonica | Film Score (Once Upon a Time in the West) | 1:06 |  |  |
| The Lively Ones | Surf Rider | Film Score (Pulp Fiction) | 1:34 |  |  |
| Clint Mansell | Lux Aeterna | Film Score (Requiem for a Dream) | 3:28 |  |  |
| John Williams | Across the Stars | Film Score (Star Wars) | 1:10 | 3:34 | 3:55 |
| John Williams | Across the Stars | Film Score (Star Wars) | 1:40 | 3:42 |  |
| Clint Mansell | Death Is The Road To Awe | Film Score (The Fountain) | 5:20 |  |  |
| John Debney | Resurrection | Film Score (The Passion of the Christ) | 0:45 |  |  |
| Hans Zimmer | Hummel gets the Rockets | Film Score (The Rock) | 4:27 |  |  |
| James Horner | My Heart will go On | Film Score (Titanic) | 2:27 |  |  |
| Crowfoot | Larks in May | Folk | 0:10 | 2:00 |  |
| Grainger | Rutherford Park Poachers | Folk | 1:40 | 3:40 |  |
| Sheefra | Da Slockit Light | Folk | 1:00 | 2:10 |  |
| The Assembly | Cheris / Fading Light | Folk | 4:01 | 6:14 |  |
| Los Angeles Guitar Quartet | Congan | Guitar | 2:09 |  |  |
| Los Angeles Guitar Quartet | Mysterious Habitats | Guitar | 1:21 | 2:20 |  |
| Pat Metheny | Make Peace | Guitar | 4:34 |  |  |
| Agustin Barrios | Julia Florida - Barcarola | Guitar - Classical | 0:56 | 1:45 | 2:07 |
| Rodrigo Y Gabriela | Diablo Rojo | Guitar - Flamenco | 2:23 |  |  |
| Rodrigo y Gabriela | Tamacun | Guitar - Flamenco | 1:58 |  |  |
| Leo Brouwer | Danza del Altiplano | Guitar - Folk | 2:11 | 2:23 | 2:51 |
| Leo Brouwer | Sonata III (La Toccata de Pasquini) | Guitar - Latin | 1:04 | 1:35 | 2:54 |
| Children of Bodom | Vivaldi's Four Seasons | Heavy Metal | 1:13 |  |  |
| Steve Vai | Beethoven's 5th | Heavy Metal | 2:00 |  |  |
| Nik Bartsch | Ronin Stoa - Modul 33 | Instrumental - Jazz | 6:25 |  |  |
| Nik Bartsch | Ronin Stoa - Modul 36 | Instrumental - Jazz | 3:00 |  |  |
| Cordame | Valse Hebraique | Instrumental - Strings | 0:35 |  |  |
| Brad Mehldau | Knives Out | Instrumental Post-Rock | 4:45 | 7:25 |  |
| Brad Mehldau | Paranoid Android | Instrumental Post-Rock | 2:32 | 6:13 |  |
| Explosions in the Sky | First Breath After Coma | Instrumental Post-Rock | 2:25 | 3:30 | 8:10 |
| Explosions in the Sky | First Breath After Coma | Instrumental Post-Rock | 3:34 | 8:27 |  |
| Joe Satriani | Always with Me, Always with You | Instrumental Rock | 2:13 |  |  |
| Torngat | Bell Duet | Instrumental Rock | 0:48 |  |  |
| Torngat | Mouton Noir | Instrumental Rock | 1:12 |  |  |
| Alizadeh | Jamedaran | International | 3:10 |  |  |
| Alizadeh | Nahoft and Faroud | International | 2:53 |  |  |
| Komitas | Varashapat | International | 0:54 |  |  |
| Bob Mintzer Big Band | Chant | Jazz | 1:35 |  |  |
| Cannonball Adderley | One for Daddy-O | Jazz | 0:40 |  |  |
| Cannonball Adderley | Work Song | Jazz | 0:52 |  |  |
| Charles Mingus | Fables of Faubus | Jazz | 0:20 | 7:10 |  |
| Chick Corea | Akoustic Band – Morning Sprite | Jazz | 1:30 |  |  |
| Chris Potter | Morning Bell | Jazz | 8:40 |  |  |
| Darcy James Argues | Ferromagnetic | Jazz | 6:25 |  |  |
| Darcy James Argues | Flux in a Box | Jazz | 3:15 |  |  |
| Dave Douglas and Nomad | North Point Memorial | Jazz | 1:58 |  |  |
| Dave Holland | Claressence | Jazz | 4:26 |  |  |
| David Binney | Out Beyond Ideas | Jazz | 3:10 |  |  |
| Jaga Jazzist | Day | Jazz | 1:15 |  |  |
| Joe Lovano Nonet | Charlie Chan | Jazz | 0:03 |  |  |
| Kenny Garrett | Equinox | Jazz | 2:35 |  |  |
| Oliver Jones | Someone to Watch over Me | Jazz | 0:11 |  |  |
| Stan Getz | Round Midnight | Jazz | 1:26 |  |  |
| Thad Jones | Walkin About | Jazz | 1:16 |  |  |
| Phish | You Enjoy Mylsef | Jazz Fusion | 10:50 |  |  |
| Joe Pass | Misty | Jazz/Blues | 2:00 |  |  |
| Philip Glass | Mishima: Stage Blood is Not Enough | Opera - Instrumental | 3:30 |  |  |
| Blonde Redhead | For the Damaged | Post-Rock | 0:35 | 1:00 |  |
| Do Make Say Think | Horns of a Rabbit | Post-Rock | 2:29 |  |  |
| Do Make Say Think | The Landlord is Dead | Post-Rock | 3:25 |  |  |
| Godspeed You! Black Emperor | Static | Post-Rock | 11:20 |  |  |
| Godspeed You! Black Emperor | Storm | Post-Rock | 3:37 |  |  |
| Transiberian Orchestra | Nutcracker Suite | Progressive Rock | 0:05 |  |  |
| Infected Mushroom | Suliman | Psychedelic Rock | 4:23 |  |  |
| Infected Mushroom | Heavyweight | Psychedelic Rock | 4:37 |  |  |
| Pink Floyd | Shine on You Crazy Diamond | Psychedelic Rock | 5:00 |  |  |
| Infected Mushroom | Special place | Psychedelic Trance | 4:01 |  |  |
| Infected Mushroom | Vicious Delicious | Psychedelic Trance | 5:50 |  |  |
| Led Zeppelin | Moby Dick | Rock | 0:32 |  |  |
| Led Zeppelin | White Summer/Black Mountain Side | Rock | 1:13 |  |  |
| Strangely Dim | Running Song | Rock | 3:50 |  |  |
| Piazzolla | Libertango | Tango | 2:51 |  |  |
| Rodriguez | La Cumparsita | Tango | 2:36 |  |  |
| Armin van Buuren | Control Freak (Sander van Doorn Remix) | Techno | 2:04 |  |  |
| Big Room Mix | Deeper Love | Techno | 1:59 |  |  |
| Jeckyll and Hyde | Freefall | Techno | 0:39 |  |  |
| Marcel Woods | Advanced | Techno | 0:08 |  |  |
| Marcel Woods | Happy Hardcore (Sandstorm by Darude) | Techno | 0:59 |  |  |
| Misjah and Tim | Access | Techno | 1:16 |  |  |
| Sander Van Doorn | Riff | Techno | 0:27 |  |  |
| Tiesto | Adagio for Strings | Techno | 3:30 | 3:50 |  |
| Tiesto | Forbidden Paradise | Techno | 3:07 |  |  |
| Tiesto | Live at Godskitched Global Gathering | Techno | 1:04 |  |  |
| Tiesto | Nyana | Techno | 1:00 | 1:30 | 3:20 |
| Tiesto | Olympic Flame | Techno | 0:30 | 1:20 | 2:05 |
| Yoji Biomehanika | Hardstyle Disco | Techno | 2:50 |  |  |
| Yoji Biomehanika | Supersound | Techno | 1:31 |  |  |
|  |  |  |  |  |  |
